# Deoxypodophyllotoxin Inhibits Non-Small Cell Lung Cancer Cell Growth by Reducing HIF-1α-Mediated Glycolysis

**DOI:** 10.3389/fonc.2021.629543

**Published:** 2021-02-24

**Authors:** Yuping Yang, Lingling Liu, Jinghui Sun, Shu Wang, Zhongyuan Yang, Honghui Li, Na Huang, Wei Zhao

**Affiliations:** ^1^ Department of Pulmonary and Critical Care Medicine, The First Affiliated Hospital of Chengdu Medical College, Chengdu, China; ^2^ School of Laboratory Medicine/Sichuan Provincial Engineering Laboratory for Prevention and Control Technology of Veterinary Drug Residue in Animal-origin Food, Chengdu Medical College, Chengdu, China; ^3^ Development and Regeneration Key Laboratory of Sichuan Province, Chengdu Medical College, Chengdu, China; ^4^ State Key Laboratory of Natural Medicines, Department of Pharmaceutics, China Pharmaceutical University, Nanjing, China; ^5^ Cancer Center, Sun Yat-Sen University, Guangzhou, China; ^6^ Department of Refractive Surgery, Chengdu Aier Eye Hospital, Chengdu, China

**Keywords:** deoxypodophyllotoxin, non-small cell lung cancer, HIF-1α, aerobic glycolysis, tumor progression

## Abstract

Cancer cell proliferation is a metabolically demanding process that requires high rate of glycolysis to support anabolic growth. Deoxypodophyllotoxin (DPT) is a natural flavonolignan with various pharmacological activities, including antitumor effect. However, whether DPT affects the metabolic reprogramming of cancer cells is unknown. The purpose of this study is to investigate the role of DPT on non-small cell lung cancer (NSCLC) and to explore whether HIF-1α-mediated glycolysis is involved in its mechanism of action.The level of HIF-1α mRNA and protein in NSCLC cells following DPT treatment was detected using qRT-PCR and western blotting, respectively. Cell Counting Kit-8 (CCK-8) and caspase-3 activity assays were performed to analyze cell proliferation and apoptosis. The underlying molecular mechanism was identified by dual luciferase assay, Western blotting, qRT-PCR, glucose consumption, lactate production, and immunoprecipitation. A murine NSCLC model was used to clarify the effect of DPT treatment on tumor cell proliferation. Our findings showed that DPT treatment inhibited NSCLC cell growth in a dose- and time-dependent manner. Further analysis suggested that DPT treatment inhibited HIF-1α signaling pathway by Parkin-mediated protein degradation in NSCLC cells. DPT treatment significantly decreased glucose consumption and lactate production. In addition, DPT treatment reduced the expression of HIF-1α target genes, including GLUT1, HK2 and LDHA, resulting in reduction in glycolysis. We further revealed that DPT-induced cell growth inhibition and increased glucose and lactate levels could be reversed by overexpressing HIF-1α. Additionally, we found that DPT repressed NSCLC growth and GLUT1, HK2 and LDHA expression *in vivo*. Overall, this study suggested that DPT inhibited NSCLC growth by preventing HIF-1α-mediated glycolysis.

## Introduction

Lung cancer is the most frequently diagnosed cancer and its incidence has been increasing in recent years (1, 2). Lung cancer remains a leading cause of cancer-associated mortality for men and women worldwide (1). The development of lung cancer is a multistep process involving alterations in oncogenes and tumor suppressor genes, and alsoother factors such as alcohol consumption, smoking, pathogenic infections and genetic factors (3–5). NSCLC accounts for over 80% of all lung cancer cases.Because the onset of NSCLC is asymptomatic,NSCLC is usually diagnosed in late clinical stages when surgical resection is not possible. The current prognosis of patients with NSCLC is very poor with a 5-year overall survival (OS) rate of only 10% (6, 7). Thus, there is an urgent need to find novel and effective means for the treatment of NSCLC.

Aerobic glycolysis, also known as the Warburg effect, was discovered almost a century ago (8–10). It has been considered as a hallmark of cancer. Another hallmark of cancer is hyper-cell proliferation. In addition to the high energy requirements of enhanced proliferation, cell division also requires large amounts of biomolecules, including nucleic acids and lipids, for which glucose is an important biosynthetic precursor. Enhanced levels of glucose transporters and enzymes in the glycolytic pathway and upregulated lactate level are often found in NSCLC cells, and inhibition of aerobic glycolysis arrests cancer cell proliferation (11–13).

Hypoxia inducible factor 1α (HIF-1α) is one of the primary glycolytic regulators. HIF-1α is ubiquitinated by the von HippelLindau protein and degraded *via* the proteasomal pathway under normoxic condition (14, 15). Due to the low oxygen content in most solid tumors, the glycolytic enzymes are consistently upregulated in cancer cells (9, 16, 17). Increased HIF-1α protein level isinduced by activated RAS, loss of p53 or increased heat shock protein 90 (Hsp90) in cancer cells regardless of the availability of oxygen (18–21). The glycolytic proteins,glucose transporter 1 (GLUT1), hexokinase 2 (HK2) and phosphoglycerate kinase 1 (PGK1) are genes that are upregulated by HIF-1α (22). Therefore, targeting HIF-1α-mediated metabolic pathways in tumor cells has been hypothesized to be a valuable therapeutic strategy.

Deoxypodophyllotoxin (DPT) is a natural flavonolignan that destabilizes microtubules and has various pharmacological activities, including anti-inflammatory, antiviral and antitumoreffects (23–25). Although there have been some studies on DPT (26, 27), the mechanism of its antitumor activity in NSCLC has not been elucidated. This study aims to investigate the potential therapeutic effect of DPT and its mechanism of action in NSCLC and to explorethe involvement of HIF-1α-mediated glycolysis.

## Materials and Methods

### Cell Culture and Drug Treatment

The human NSCLC cell lines A549, SK-MES-1, H460, and SPC-A1 were obtained from the Cell Bank of Type Culture Collection of Chinese Academy of Sciences (Shanghai, China) and were cultured in Dulbecco’s modified Eagle’s medium (DMEM, Gibco, Waltham, MA) supplemented with 1% penicillin-streptomycin at 37°C with 5% CO_2_and 21% O2. Cells were treated with different concentrations (0, 4, 8, 12, 16, and 20 nM) of DPT for 12, 24, and 48 h. Then, the cells were subjected to the following experiments.

### Cell Transfection

For transfection, 2 × 10^4^ A549 or SK-MES-1 cells were seeded in each well of a 24-well plate. After 24 h, the cells were transfected with the control or HIF-1α overexpressing plasmid (pcDNA-HA-HIF-1α, MobaiBiotech., Nanjing, China) using Lipofectamine 3000 (Thermo Fisher Scientific, Waltham, MA) according to the manufacturer’s instructions. Cells were harvested for analysis after 48 h. DPT (16 nM) was added to the medium 48 hbefore the experiment.

### CCK-8 Assay

Cell viability was assessed by the Cell Counting Kit-8 (CCK-8) assay (DOJINDO, Kumamoto, Japan) at 12, 24, and 48 h of drug treatment. Cells (5 × 10^3^ cells/well) were seeded in 96-well plates and incubated with different concentrations (0, 4, 8, 12, 16, and 20 nM) of DPT for 12, 24, and 48 h. Then, 10 µl of CCK-8 assay solution was added to each well and incubated for another 4 h. Finally, the wells were washed with PBS, dried, and 150 μl of DMSO was added. Light absorbance was measured by a microplate reader at 490 nm (Thermo Fisher Scientific).

### Caspase-3 Activity Assay

NSCLC cells were cultured in 96-well plates. After DPT treatment for 48 h in DMEM supplemented with 10% FBS, caspase-3 activity was measured. A caspase-3 activity assay kit (Beyotime, China)was used for the measurement of caspase-3 enzymatic activity. Briefly, 50 μl of cell lysis buffer was prepared by mixing 10 μl Ac-DEVD-pNA (2 mM) and 40 μl buffer and was loaded into a 96-well plate. After incubation at 37°C for 4 h, the light absorbance was measured at 405 nm by a microplate reader (Thermo Fisher Scientific). The caspase-3 activity in each sample solution was calculated by the standard curve method. The final results were normalized to the quantity of total protein using a Bradford protein quantitative kit (Beyotime).

### Dual Luciferase Assay

After DPT treatment, the Cignal Finder Cancer 10-Pathway Reporter Array (Qiagen, Shanghai, China; Cat. No.: 336821) was used to characterize the signaling pathways that were altered in NSCLC cells as described previously (28). Then, luciferase assays were performed to confirm the prediction. Briefly, 1 × 10^5^ A549 or SK-MES-1 cells were transfected using Lipofectamine 3000 (Thermo Fisher Scientific) with 0.2 µg of triple hypoxia response element (3xHRE). A renilla luciferase plasmid was cotransfected as a transfection efficiency control. After 24 h, the cells were harvested, and luciferase activity was measured using the Dual-Luciferase Reporter Assay System (Promega, Madison, WI, USA) according to the manufacturer’s protocol.

### Real-Time Quantitative PCR

Total RNA from cells and tissues was extracted using TRIzol reagents (Invitrogen, Carlsbad, CA, USA). RNA was reverse transcribed into cDNA using the Reverse Transcription System Kit (Promega). The reaction mixture was as follows: SYBR Premix Ex Taq II (Bio-Rad, Hercules, CA, USA), 2 μl of cDNA, 5 μl of 2× master mix, 0.5 μl forward/reverse primer and 2 μl of pure water. RT-PCR was performed on an ABI7900HT machine (Applied Biosystems, Foster City, CA) with 3 replicates. Amplification conditions were 95°C for 5 min, followed by 40 cycles of 95°C for 15 s and 60°C for 1 min. Primers were obtained from GeneScript (Nanjing, China), and their sequences were as follows: HIF-1α: Forward 5′-TTGCTCATCAGTTGCCACTTCC-3′, Reverse 5′-AGCAATTCATCTGTGCTTTCATGTC-3′; CAIX: Forward 5′-GGATCTACCTACTGTTGAGGCT-3′, Reverse 5′-CATAGCGCCAATGACTCTGGT-3′; GLUT1: Forward 5′-GATTGGCTCCTTCTCTGTGG-3′, Reverse 5′-TCAAAGGACTTGCCCAGTTT-3′; HK2: Forward 5′-GAGCCACCACTCACCCTACT-3′, Reverse 5′-CCAGGCATTCGGCAATGTG-3′; LDHA: Forward 5′-AAGCGGTTGCAATCTGGATTCAG-3′, Reverse 5′-GGTGAACTCCCAGCCTTTCC-3′; and GAPDH: Forward 5′-TGACGTGGACATCCGCAAAG-3′, Reverse 5′-CTGGAAGGTGGACAGCGAGG-3′. Data were calculated by the 2^-ΔΔCq^ method after normalization to β-actin mRNA level.

### Western Blot Analysis

Cells were washed with PBS and homogenized in RIPA buffer (Roche, Basel, Switzerland). Proteins were separated by 10% SDS-PAGE (Roche) and transferred to a polyvinylidene fluoride membrane (Millipore, Burlington, MA). Membranes were blocked with 5% skim milk in TBST (TBS with 20% Tween-20) for 2 h at room temperature before incubation with primary antibodies against β-actin (Cell Signaling Technology, Cat. No. 4970; 1:1000), FLAG (Proteintech, Cat. No.66008-3; 1:1,000), p21 (Proteintech, Cat. No. 66214-1; 1:1,000), and HIF-1α (Cell Signaling Technology, Cat. No. 36169; 1:1,000) at 4°C overnight. Afterward, the membranes were incubated with a secondary antibody (Cell Signaling Technology, Cat. No. 7074; 1:5,000) for 1 h at room temperature. Bands were visualized by SuperSignalWest Pico Chemiluminescent Substrate (Thermo Fisher). β-actin was used as an internal loading control.

### *In vitro* Ubiquitination Assay

For *in vitro* ubiquitination assays, NSCLC cells were transfected with pcDNA-HIF-1α (MobaiBiotech.) and the transfected cells were treated with 16 nM DPT for 48 h. Cell lysates were harvested and HIF-1αwas purified by immunoprecipitation. Purified HIF-1α protein (0.4 μg) was incubated with reaction mixtures (50 μl) consisting of buffer [5 mM MgCl_2_, 50 mM Tris (pH 7.4), 1 mM DTT, and 2 mM ATP], 0.5 µg of E1 (Boston Biochem, Cambridge, MA, USA), 0.5 µg of E2 (UbcH7; Boston Biochem), 5 µg of Ub (Boston Biochem), and 0.5 µg of PINK1 (Boston Biochem). After incubation for 3 h at 37°C, the postreaction mixtures were used for Western blot analysis with an anti-Ub antibody (sc-8017, Santa Cruz Biotechnology, TX, USA; 1:2,000).

### Immunoprecipitation

Immunoprecipitation assay was performed as described previously. A549 cells were transfected with pcDNA-FLAG-Parkin(MobioTech.) for 24 h with or without DPT (16 nM DPT) and lysed in EBC buffer for 30 min. The precleared soluble supernatants were centrifuged at 12,000 × g for 20 min at 4°C to remove the debris. The lysates were mixed with 1 g of anti-FLAG (Proteintech, Cat. No. 66008-3) antibody for 16 h at 4°C, followed by precipitation of the protein A/G-agarose beads. After washing the immune complexes, the bound proteins were resuspended in sodium dodecyl sulfate sample buffer, separatedby sodium dodecyl sulfate-polyacrylamide gel electrophoresis, and incubated with antibodies to HIF-1α, p21, or FLAG. The elution was analyzed by Western blotting.

### Glucose Consumption and Lactate Production Measurements

To determine whether DPT treatment altered glucose utilization or lactate production in A549 and SK-MES-1 cells, cells were plated at 2×10^5^ cells per flask and incubated at 37°C with 5% CO_2_. After allowing the cells to attach and grow for 24 hr, 16 nM DPT was added to the flasks and incubated at 37°C for 48 hr. The medium was collected by centrifugation to remove the cells, and glucose and lactate levels were detected by standard colorimetric assay kits for glucose (K606-100, BioVision, Milpitas, CA) and lactate (K607-100, BioVision) per the manufacturer’s instructions. Glucose consumption and lactate production was calculated as described in the previous report (22).

### Tumor Xenograft Assay

All animal experiments were approved by the Animal Care and Use Committee of Chengdu Medical College (No. 19JY751). Male athymic nude mice(BALB/c^nu/nu^) (6–7 weeks old) were purchased from the Model Animal Research Center(Nanjing, China). To generate subcutaneous tumors, 4 × 10^6^A549 cells were subcutaneously injected i nto the flanks of the mice. Tumor sizes (calculated by the formula volume = length × width^2^/2) and mouse weights were measured three times per week using a caliper and an electronic balancer. The animals were randomly divided into a vehicle control group and DPT treatment groups (10 or 20 mg/kg DPT, administered three times a week by intravenous injection).

### Statistical Analysis

All experiments were performed at least three times. Statistical analyses were performed with SPSS software version 18.0 (IBM Corp., Armonk, NY). Data are displayed as the mean ± standard deviation (SD). Statistical comparisons between groups were made by one-way analysis of variance with Tukey’s *post hoc* test or Student’s t-test. A p value < 0.05 was considered statistically significant.

## Results

### DPT Dose- and Time-Dependently Inhibited the Proliferation and Promoted Apoptosis of Lung Cancer Cells

**Figure 1:**
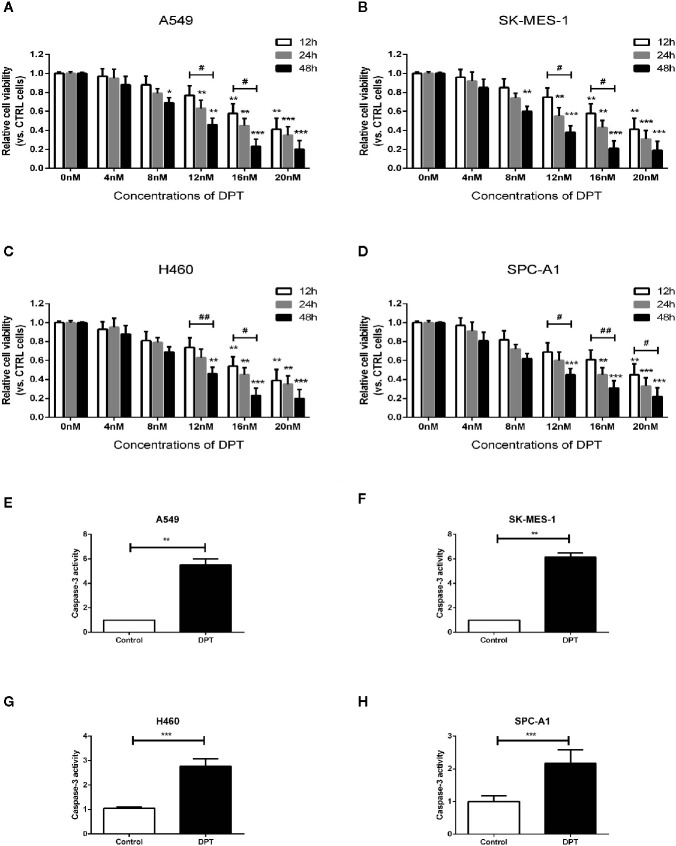
DPT dose- and time-dependently inhibited the proliferation and promoted apoptosis of lung cancer cells. **(A–D)** A549, SK-MES-1, H460 and SPC-A1 cells were treated with different concentrations (0, 4, 8, 12, 16, and 20 nM) of DPT for varying lengths of time (12, 24 and 48 h). Cell viability was detected by CCK-8 assay. **(E–H)** Apoptosis of A549 and SK-MES-1 cells following treatment with 16 nM DPT for 48 h,detected using acaspase-3 activity detection kit. *P < 0.05, **P < 0.01, ***P < 0.01, compared with the control group; ^#^P < 0.05. ^##^P < 0.01, compared as indicated.

To assess the effect of DPT on lung cancer cells, A549, SK-MES-1, H460 and SPC-A1 cellswere treated with various concentrations of DPT for the indicated time periods, and CCK-8 assays were performed to measure cell viability. As shown in **Figures 1A–D**, DPT suppressed cell viability in a dose- and time-dependent manner in A549, SK-MES-1, H460, and SPC-A1 cells. To further investigate the mechanisms of DPT toxicity, we assessed apoptosis levels in these NSCLC cells following treatment with 16 nM DPT for 48 h. Caspase-3 activity was detected and found increased in the DPT-treated cells compared with the control cells (**Figures 1E–H**). These data indicated that DPT can inhibit cell proliferation and promote apoptosis of lung cancer cells.

### DPT Decreased Glycolysis in A549 and SK-MES-1 Cells

**Figure 2:**
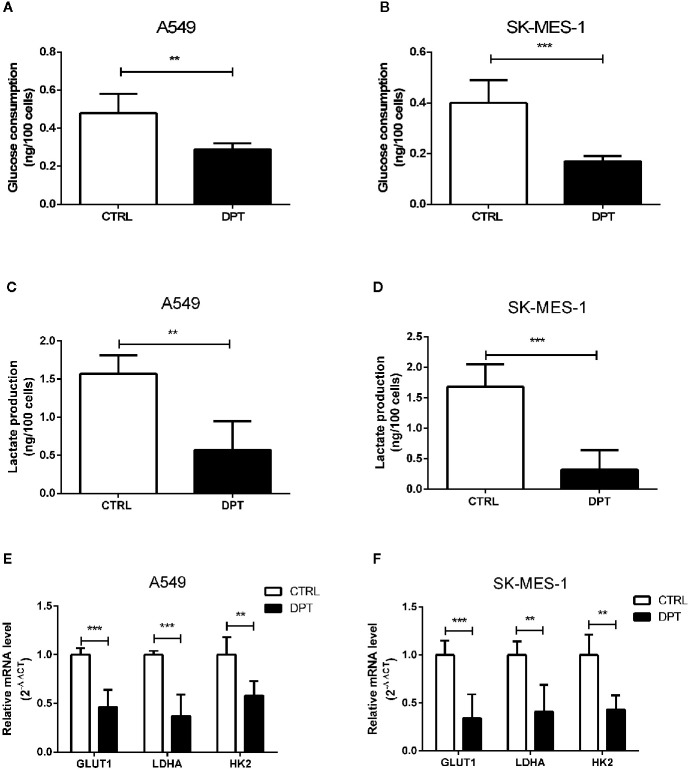
DPT reduced glycolysis in A549 and SK-MES-1 cells. A549 and SK-MES-1 cells were treated with 16 nM DPT for 48 h. **(A–D)** Glucose consumption and lactate production detected using specific test kits. The mRNA levels of the glycolytic pathway-related genes including GLUT1, LDHA and HK2 in A549 **(E)** and SK-MES-1 cells **(F)** detected by qRT-PCR. **P < 0.01, ***P < 0.001.

Metabolic pathways in cancer cells are usually reprogrammed to favor glycolysis, which provides a source of metabolic intermediates needed for cell proliferation (29). A549 and SK-MES-1 lung cancer cells treated with 16 nM DPT showed reduced glucose consumption and lactate production compared with the control cells (**Figures 2A–D**). Furthermore, the mRNA levels of glycolytic pathway genes, including GLUT1, lactate dehydrogenase A (LDHA), and HK2, were significantly decreased in A549 and SK-MES-1 cells following 48 h of DPT treatment (**Figures 2E, F**).

### DPT Blocked HIF-1α Signaling in A549 and SK-MES-1 Cells

**Figure 3:**
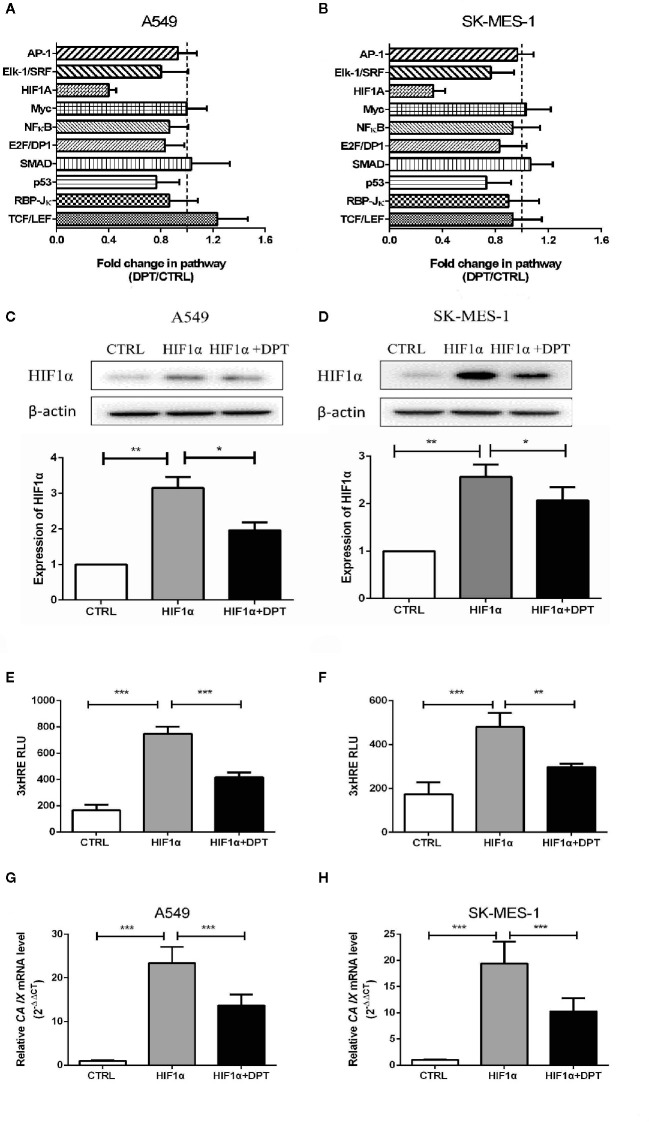
DPT inhibited HIF-1α signaling in A549 and SK-MES-1 cells. A549 **(A)** and SK-MES-1 **(B)** cells were treated with 16 nM DPT for 48 h. The various signaling pathways were analyzed using the Cignal Finder Cancer 10-Pathway Reporter Array. A549 **(C)** and SK-MES-1 **(D)** cells were transfected with a HIF-1α expression plasmid in the presence or absence of 16 nM DPT for 48 h. Western blot assay detected HIF-1α protein and quantified by ImageJ. **(E, F)** HIF-1α luciferase activity detected using luciferase reporter assay. **(G, H)** mRNA expression of *CA IX* in A549and SK-MES-1 cells detected by qRT-PCR. *P < 0.05,**P < 0.01, ***P < 0.001.

To further characterize the mechanism by which DPT inhibits A549 and SK-MES-1 cell growth, we performed an analysis on various signaling pathways using Qiagen Cignal Finder. It was found that DPT significantly inhibited HIF-1α signaling in both lung cancer cell lines, indicating impairment ofHIF-1α-dependent signaling (**Figures 3A, B**). To examine whether DPT treatment affected HIF-1α protein levels, A549 and SK-MES-1 cells were transfected with pcDNA-HIF-1α (**Figures 3C, D**) in the presence or absence of DPT for 48 h. As shown in **Figures 3E, F**, DPT significantly decreased HIF-1α-dependent luciferase activity following hypoxia treatment in both cell lines.Furthermore, we examined the expression of*CA IX*, which is a specific target gene of HIF-1α and is a key mediator of tumor progression (30).As shown in **Figures 3G, H**, DPT treatment decreased *CA IX* mRNA expression compared with the control treatment.

### DPT Promoted HIF-1α Ubiquitination and Degradation by Increasing the Interaction Between HIF-1α and Parkin

**Figure 4:**
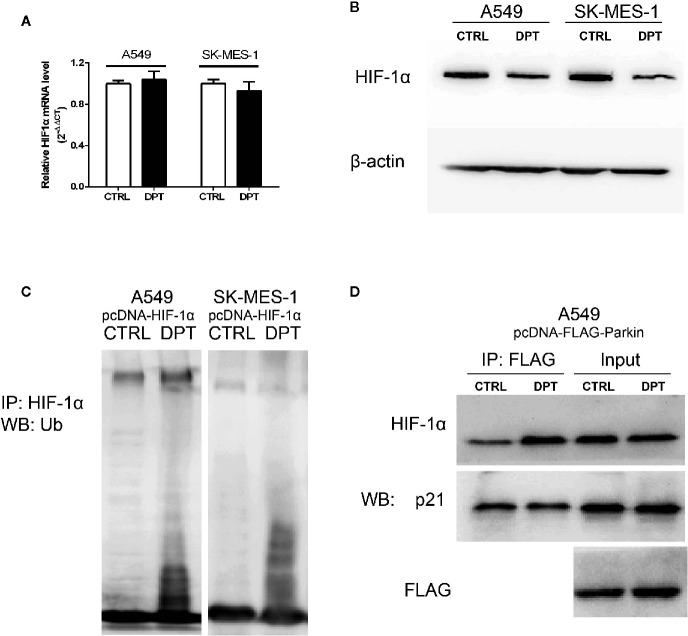
DPT promoted HIF-1α ubiquitination and degradation by increasing the interaction between HIF-1α and Parkin. A549 and SK-MES-1 cells (transfected with pcDNA-HIF-1α) treated with 16 nM DPT for 48 hr. **(A, B)** HIF-1α mRNA detected by qRT-PCR, and HIF-1α protein detected by Western blot. **(C)** *In vitro* ubiquitination assay detected the effect of DPT on HIF-1αubiquitination in A549and SK-MES-1 cells. **(D)** Immunoprecipitation detected the interaction of Parkin with HIF-1α and p21.

As shown in **Figures 4A, B**, DPT decreased HIF-1α protein level but had no effect on the mRNA levels. To investigate whether DPT negatively regulates HIF-1α through ubiquitin-proteasome degradation, we examined HIF-1α degradation after DPT treatment. In NSCLC cells transfected with pcDNA-HIF-1α, DPT decreased HIF-1α protein level and increased the ubiquitinationlevel (**Figure 4C**). These data indicatethat DPT negatively regulates HIF-1α through ubiquitin-proteasome degradation. Bioinformatic analysis suggested that HIF-1α is the potential targets of DPT (**Supplementary Data**), and the immunoprecipitation results demonstrated that DPT treatment promoted the interaction of HIF-1α with Parkin, which is a HIF-1α interacted E3 ligase (31) (**Figure 4D**), but not p21, which is another substrate of Parkin (32). These data demonstrated that DPT targets HIF-1α and increases the interaction between HIF-1α and Parkin, followed by HIF-1α degradation.

### DPT Inhibited HIF-1α Overexpression-Induced Cell Growth and Glycolysis

**Figure 5:**
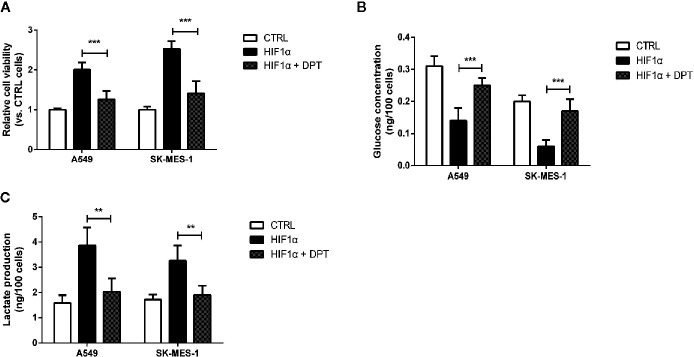
DPT inhibited HIF-1α-induced cell growth and glycolysis.A549 and SK-MES-1 cells treated with 16 nM DPT and the HIF-1α plasmid for 48 h. **(A)** Cell viability detected by CCK-8 assay. **(B, C)** Glucose consumption and lactate production detected using specific test kits. **P < 0.01, ***P < 0.001.

To confirm whether DPT-induced reduction in cell growth and glycolytic flux is mediated through inhibition of HIF-1α signaling, A549 and SK-MES-1 cells were treated with 16 nM DPT for 48 h after transfection with a HIF-1α expression plasmid. HIF-1α overexpression increased cell viability, glucose consumption and lactate production, all of which could be reversed by DPT treatment (**Figures 5A–C**). These data indicated that HIF-1α is required for the DPT-induced inhibition of cell growth and glycolysis.

### DPT Inhibited A549 Xenograft Growth *In Vivo*

**Figure 6:**
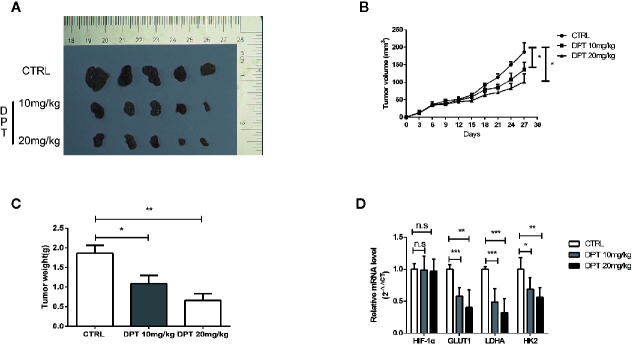
DPT inhibited A549 xenograft growth *in vivo*. Mice were randomly divided into the vehicle control group and DPT treatment groups (10 or20 mg/kg DPT). **(A, B)** *In vivo* A549 tumor and control xenograft growth curve. Tumor sizes were measured three times per week with a calliper and calculated using the formula volume = (length×width^2^)/2. **(C)** The average tumor weight of the DPT treatment (10 or 20 mg/kg) and control xenografts at the end point. **(D)** The mRNAlevel of HIF-1α and glycolytic pathway-related genes including GLUT1, LDHA, and HK2 detected by qRT-PCR. *P < 0.01, **P < 0.01, ***P < 0.001, n.s., not significant.

We further examined the effect of DPT on HIF-1α and glycolysis in A549 xenografts in nude mice. The mice were randomly divided into three groups with 5 animals in each group. As shown in **Figures 6A–C**, DPT (10 mg/kg or 20 mg/kg) significantly reduced xenograft volumes and weights. Additionally, DPT (10 or 20 mg/kg) decreased GLUT1, LDHA and HK2 mRNA levels compared with the control treatment (**Figure 6D**). These findings indicated that DPT inhibited tumor growth and glycolysis *in vivo*.

## Discussion

This study investigated the antitumoreffect and potential mechanism of action of DPT in NSCLC both *in vitro* and *in vivo*. The data revealed that DPT has a potent growth-inhibitory effect onA549 and SK-MES-1 cells. Additionally, DPT inhibited the growth of A549 xenograft *in vivo*. These findings indicated that DPT may be a potential drug for NSCLC treatment. Furthermore, our data showed that DPT functions as an anticancer agent in NSCLC by increasing HIF-1α degradation to reduce glycolysis. Here, we showed that DPT suppresses HIF-1α activation at the protein level in NSCLC cells. Moreover, based on the structure of DPT, two state-of-the-art computational methods (33, 34) were employed to predict its potential targets, and HIF-1α was one of the most representedt argets. Immunoprecipitation indicated that DPT increased the interaction between HIF-1α and Parkin, and the E3 ubiquitin ligase promoted HIF-1α protein ubiquitination and degradation.

The role of DPT has been investigated in multiple cancers, including breast cancer (35), osteosarcoma (23), gastric cancer (36) and NSCLC (37). The findings suggested that DPT has a wide range of effect on tumor development, such as inhibiting tumor growth, inducing cell cycle arrest, inhibiting angiogenesis and promoting apoptosis. A previous study also reported that DPT triggers necroptosis in human NSCLC NCI-H460 cells (37). To assess the potential antitumor effect of DPT, this study investigated its time- and dose-dependent activity on lung carcinoma cell lines. Our data demonstrated that DPT inhibited cell viability and induced apoptosis. Additionally, Qiagen Cignal Finder was used to predict the signaling pathways that were altered following DPT treatment by which we found that HIF-1α was a DPT target.

HIF-1α is degraded under normoxic condition *via* the proteasome pathway but is stabilized under hypoxia (38).To date, a series of studies have reported that HIF-1α expression could be an important predictor of tumor prognosis, including for hepatocellular carcinoma, cervical carcinoma and lung cancer (39–41). A previous study also reported that HIF-1α is overexpressed in NSCLC and that targeting the HIF pathway may be a promising approach for NSCLC management (42). In other reports, DPT has been shown to inhibit cancer cell cycle/microtubule formation and induce apoptosis/autophagy both *in vitro* and *in vivo* (23, 43–46). Our study supports previous data showing that DPT acts as an anticancer agent through degradation of HIF-1α.

HIF-1α is a master transcriptional regulator of glycolysis that controls the expression of amultitude of glycolytic genes, including HK2, GLUT1, PGM, PGK1 and LDHA (38). Cancer cells exhibit significant alterations in glucose metabolism compared with normal cells (47). Previous studies have indicated that cancer cells use aerobic glycolysis (or Warburg metabolism) to facilitate cell proliferation *by* providing sufficient metabolic intermediates (48). Although some studies have shown that DPT triggers necroptosis in human NSCLC NCI-H460 cells (37), whether DPT inhibits glycolysis in NSCLC cells is unclear. In this study, we demonstrated that HIF-1α overexpression increased glucose consumption and induced lactate production, both of them could be reversed by DPT treatment. Additionally, DPT decreased the expression of genes in the glycolytic pathway. To exclude mycoplasma infection, which was found to cause major shifts in cellular metabolism (49), mycoplasma testing was performed on the cell lines utilized in this study.

As both HIF-1α and DPT have been reported to beassociated with cancer cell cycle progression (43, 50), cancer cell microtubule destabilization (45, 51), cancer cell necroptosis (37, 52) and autophagy (23, 53), our data suggest that the effect of DPT on cancer cells might occur through the degradation of HIF-1α. Whether DPT inhibits the NSCLC cell cycle progression, microtubule destabilization, necroptosis and autophagy by abrogating HIF-1α requires further study.

Taken together, our study demonstrated that DPT inhibitscell proliferation of NSCLC by inhibiting glycolysis *via* downregulation of HIF-1α expression. Our*in vivo* experiments supported the notion that DPT could be a potential candidate for NSCLC therapy.

## Data Availability Statement

The original contributions presented in the study are included in the article/**Supplementary Material**. Further inquiries can be directed to the corresponding authors.

## Ethics Statement

The animal study was reviewed and approved by the Animal Care and Use Committee of Chengdu Medical College.

## Author Contributions

Conceptualization,WZ. Methodology, YY, LL, and JS. Validation, YY, LL, SW, and JS. Formal analysis, NH and HL. Investigation, NH, YY, and WZ. Resources, HL. Data, YY and LL. Writing—original draft preparation, NH and WZ. Writing—review and editing, YY, WZ, and NH. Supervision, NH and WZ. Funding acquisition, NH and WZ. All authors contributed to the article and approved the submitted version.

## Funding

This work was supported by the National Natural Science Foundation of China (81602636), Key project of science of Sichuan Education Department (18ZA0164), Natural Science Foundation of Chengdu Medical College (CYZ18-04), National Key Clinical Specialty Training Program of The First Affiliated Hospital of Chengdu Medical College (CYFY2018GLPHX02), Special key fund of The First Affiliated Hospital of Chengdu Medical College (CYFY2019ZD02), Research Fund of Development and Regeneration Key Laboratory of Sichuan Province (SYS18-08), Scientific Research Project of Sichuan Provincial Administration of Traditional Chinese Medicine (2020JC0021) and Miaozi project of science and Technology Department of Sichuan Province (2018RZ0095).

## Conflict of Interest

The authors declare that the research was conducted in the absence of any commercial or financial relationships that could be construed as a potential conflict of interest.

The reviewer LZ declared a shared affiliation, with no collaboration, with one of the authors ZY to the handling editor at the time of the review.
