# Supplementary material for: Deoxypodophyllotoxin Inhibits Non-Small Cell Lung Cancer Cell Growth by Reducing HIF-1α-Mediated Glycolysis
Source: Front Oncol. 2021 Feb 24;11:629543. doi: 10.3389/fonc.2021.629543 (PMC7959795; doi:10.3389/fonc.2021.629543)
Supplement: Supplementary file 1 [file DataSheet_1.pdf]

Supplementary Materials

Materialsand Methods:

Prediction of DPT's targets

To find the real *in vivo* targets of a bioactive compound, the ligand structure similarity-based method, to predict the potential targets of DPT. Based on the structure of DPT, two state-of-the-art ligand similarity-based computational methods (1, 2) were both employed to predict its potential targets. Consideration of 3D shape matching for ligand-target molecular recognition, one of the two methods we used combined both ligand 2D fingerprint similarity and 3D shape similarity to improve target prediction performance (1). To avoid any potential bias of the method, Similarity Ensemble Approach (SEA) was used as a complementary strategy (2). After got the possible targets predicted by these two methods, we filtered them based the biology we understood. Any biological data available for DPT close analogs, of which the similarity is above 90%, were collected from ChEMBL database (3). The proteins had been tested for DPT close analogs in ChEMBL database could suggest the potential targets for DPT. Furthermore, if these predicted targets have available X-ray complex structures in Protein Data Bank (4), whether there has enough space in the binding site for DPT was also be checked.

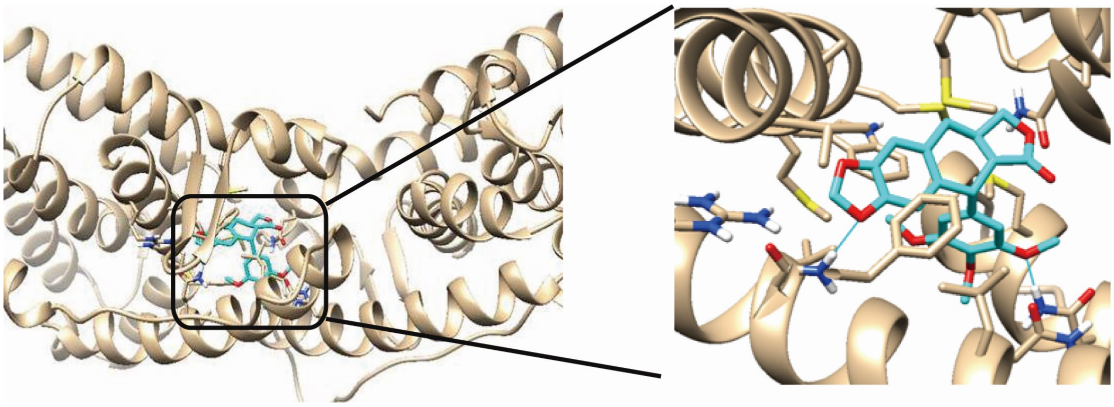

Supplementary figure Predicted Interactions between DPT and HIF-1α

Reference

1. Gfeller D, Michielin O, Zoete V. Shaping the interaction landscape of bioactive molecules. *Bioinformatics* (2013) 29(23):3073-9. doi: 10.1093/bioinformatics/btt540. PubMed PMID: 24048355.
2. Keiser M, Roth B, Armbruster B, Ernsberger P, Irwin J, Shoichet B. Relating protein pharmacology by ligand chemistry. *Nature biotechnology* (2007) 25(2):197-206. doi: 10.1038/nbt1284. PubMed PMID: 17287757.
3. Gaulton A, Hersey A, Nowotka M, Bento A, Chambers J, Mendez D, et al. The ChEMBL database in 2017. *Nucleic acids research* (2017) 45(D1):D945-D54. doi: 10.1093/nar/gkw1074. PubMed PMID: 27899562; PubMed Central PMCID: PMC5210557.

4. Rose P, Prlic A, Altunkaya A, Bi C, Bradley A, Christie C, et al. The RCSB protein data bank: integrative view of protein, gene and 3D structural information. *Nucleic acids research* (2017) 45(D1):D271-D81. doi: 10.1093/nar/gkw1000. PubMed PMID: 27794042; PubMed Central PMCID: PMC5210513.
